# Plant (di)terpenoid evolution: from pigments to hormones and beyond†

**DOI:** 10.1039/d2np00054g

**Published:** 2022-12-06

**Authors:** Zhibiao Wang, David R. Nelson, Juan Zhang, Xiangyuan Wan, Reuben J. Peters

**Affiliations:** a School of Life Sciences, Beijing University of Chinese Medicine Beijing 100029 China; b Roy J. Carver Department of Biochemistry, Biophysics & Molecular Biology, Iowa State University Ames IA 50014 USA rjpeters@iastate.edu; c Department of Microbiology, Immunology and Biochemistry, University of Tennessee Health Science Center Memphis TN 38163 USA; d Zhongzhi International Institute of Agricultural Biosciences, Shunde Innovation School, Research Center of Biology and Agriculture, University of Science and Technology Beijing Beijing 100024 China juanz@ustb.edu.cn wanxiangyuan@ustb.edu.cn

## Abstract

Covering: up to 2014–2022.

Diterpenoid biosynthesis in plants builds on the necessary production of (*E*,*E*,*E*)-geranylgeranyl diphosphate (GGPP) for photosynthetic pigment production, with diterpenoid biosynthesis arising very early in land plant evolution, enabling stockpiling of the extensive arsenal of (di)terpenoid natural products currently observed in this kingdom. This review will build upon that previously published in the Annual Review of Plant Biology, with a stronger focus on enzyme structure–function relationships, as well as additional insights into the evolution of (di)terpenoid metabolism since generated.

## Abstract 2

Diterpenoid biosynthesis in plants is derived from photosynthetic pigment metabolism, and arose early in land plant evolution, enabling stockpiling of the extensive arsenal of (di)terpenoid natural products currently observed in this kingdom.

## Graphical abstract

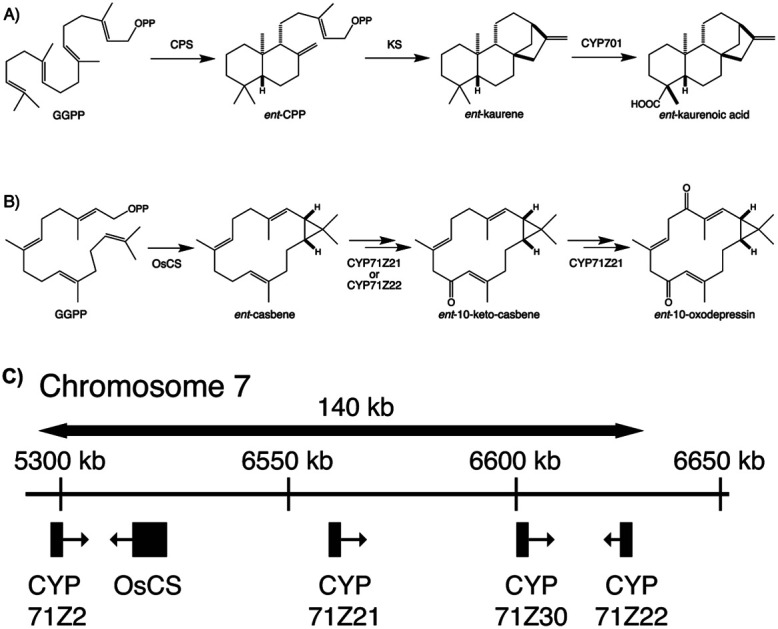

## 1 Introduction

GGPP can be considered a central metabolite in photosynthetic organisms, as it is required for production of light absorbing pigments (*i.e.*, the lipophilic phytyl side-chain of chlorophyll as well as the accessory carotenoids). However, GGPP also serves as general precursor to more specific diterpenoid biosynthesis where its constituent four isoprenyl units form the core of the derived natural product. The first appearance of such metabolism in plants appears to have been for phytohormone biosynthesis, but has since undergone dramatic expansion. Accordingly, the vast majority of the over 12 000 currently known diterpenoids are produced by this kingdom.^1^ As befitting their origins from photosynthetic pigments *in planta* diterpenoid biosynthesis is initiated in plastids, including proplastids and leucoplasts as well as chloroplasts.^2,3^ Hence, the relevant isoprenyl units are generally derived from the plastid-localized 2-C-methyl-d-erythritol 4-phosphate (MEP)-dependent isoprenoid pathway.^4^ Given its even more central role in metabolism that pathway will not be further discussed here. This review focuses on evolution of the enzymatic families that operate more selectively in (di)terpenoid metabolism, most notably those for plant terpene synthases (TPSs) as well as cytochromes P450 (CYPs) that insert oxygen into the TPS-derived hydrocarbon backbones. Although less is known about subsequently acting enzymes, which generally decorate the functional groups introduced by CYPs, these also are reviewed, as is the presence of diterpenoid biosynthetic gene clusters. In each case, this review emphasizes the additional insights gained since publication of the previous review.^1^

**Fig. 1:**
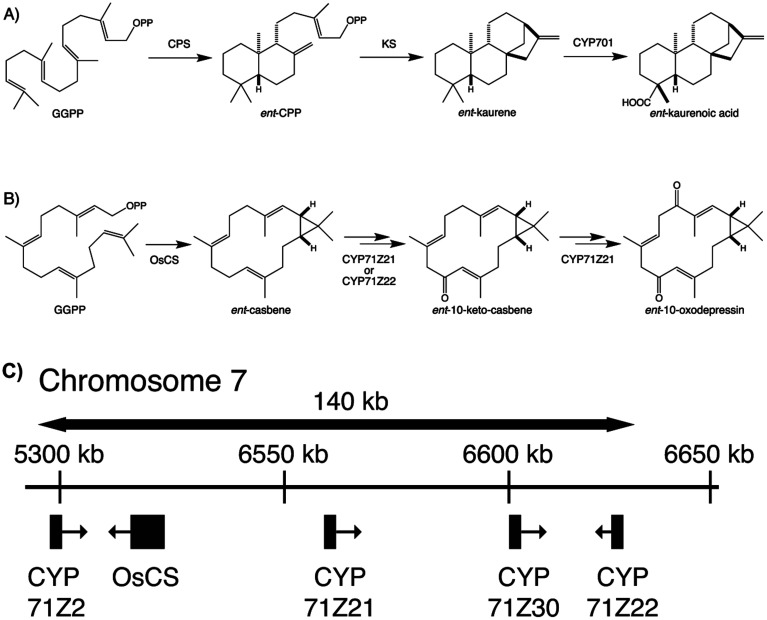
Examples of diterpenoid biosynthesis from (A) phytohormone (*ent*-kaurenoic acid) and (B) more specialized (10-oxodepressin phytoalexin) metabolism, including (C) the biosynthetic gene cluster associated with the latter from the rice genome. While both initiated from the general precursor GGPP, these provide examples of labdane-related diterpenoids, as defined by the initial activity of a class II diterpene cyclase (phytohormone) or direct cyclization by a class I diterpene synthase, as well as cytochromes P450 (CYPs) inserting oxygen(s) into the resulting hydrocarbon backbones.

To provide context two examples of diterpenoid metabolism are presented here (Fig. 1). The first represents biosynthesis of the ancestral diterpenoid phytohormones, specifically the universal production of *ent*-kaurenoic acid in such metabolism.^5^ This further provides an example of the large labdane-related diterpenoid super-family (∼7000 currently known), whose production is characterized by initial bicyclization catalyzed by class II diterpene cyclases.^6^ In this case the relevant enzyme produces *ent*-copalyl diphosphate (*ent*-CPP), whose labdane backbone inspired this nomenclature. By contrast, the biosynthesis of other diterpenoids is initiated by class I TPSs that act directly on GGPP and necessarily function in more specialized metabolism. For example, in biosynthesis of the rice phytoalexin 10-oxodepressin, whose production further provides an example of biosynthetic gene cluster assembly, as also depicted here.^7,8^

## 2 Precursors: *cis* as well as *trans*

**Scheme 1:**
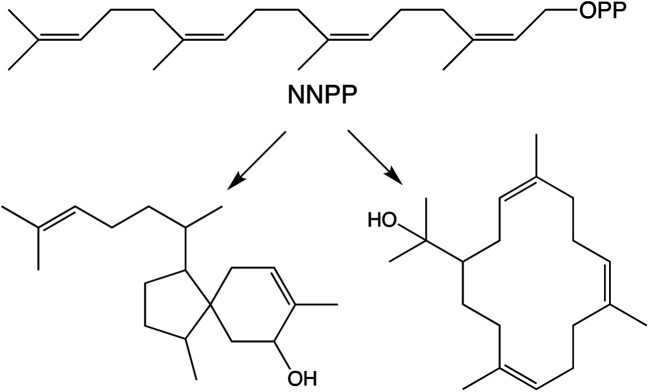
NNPP and derived diterpenes.^10^

While generally derived from the all-*trans* GGPP it has been shown that diterpenoid biosynthesis also can proceed from the all-*cis* (*Z*,*Z*,*Z*)-nerylneryl diphosphate (NNPP). At the time of the previous review only a single example from the *Solanum* genus was known.^9^ More recently NNPP-derived diterpenoids have been reported from other plant species as well (Scheme 1).^10,11^ In each case NNPP is produced by a *cis*-prenyl transferase (CPT) family member. While CPTs more typically produce long-chain isoprenoids, those forming NNPP fall within a phylogenetically distinct clade of short-chain (≤4 isoprenyl units) producing CPTs regardless of species. This indicates that use of such all-*cis* isoprenyl diphosphates, presumably as alternative terpenoid precursors, arose before separation of the relevant genera, all of which fall within the Asterid clade of the Eudicots. However, examination of a wider range of species will be required to more precisely determine when the underlying CPT gene duplication and neofunctionalization to production of shorter-chain isoprenyl diphosphate precursors arose, as well as any subsequent gene loss, along with the possibility of independent evolution of such functionality in other plant lineages.

By contrast, GGPP is produced by members of a distinct family of isoprenyl diphosphate synthases (IDSs), which gave rise to the more prototypical all-*trans* terpenoid precursors. These seem to have given rise to terpene synthases, leading to their common designation as class I enzymes. Briefly, this was first suggested by homology between their divalent magnesium (Mg^2+^) binding regions (a pair of DDxxD motifs in IDSs), which was further consistent with use of these co-factors to ionize allylic diphosphate ester bonds to initiate carbocation-based reactions in both cases, and latter supported by the observation of structural homology between the catalytically relevant domain in each family.^12^ This helical-bundle fold has been termed the α domain.^13^ Although the *IDS* gene duplication and neofunctionalization event giving rise to terpene synthases clearly occurred well before the origin of land plants, examples of more recent diversion of IDSs to terpene synthase activity also have been reported.^14^

**Fig. 2:**
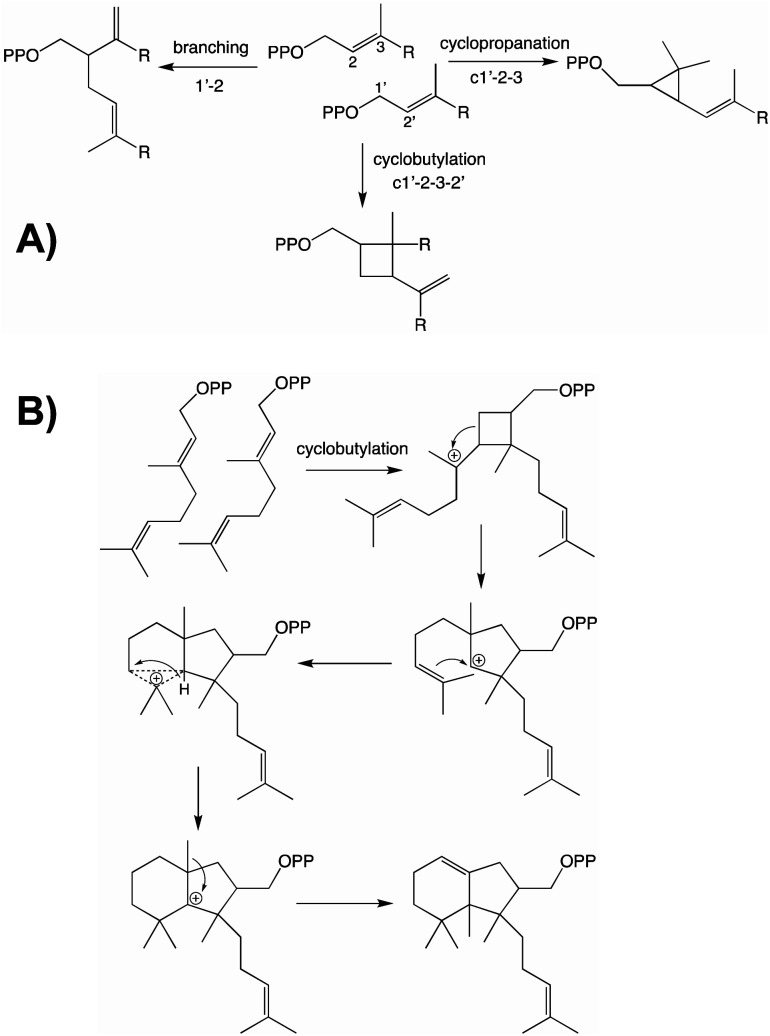
Irregular terpenoids. (A) Relevant branching, cylopropyl and cyclobutyl reactions.^22^ (B) Diterpene cyclization *via* irregular coupling.^21^

From both the CPT and IDS families examples of enzymes diverging from the canonical head to tail elongation reaction to produce irregular terpenoids *via* condensation forming alternative branching, cyclopropyl or cyclobutyl linkages have been found in plants (Fig. 2A).^15–17^ Although only a single example is found from the CPT family, the two from the IDS family are homologous and their relationship suggests these originated before diversification of the Asteraceae plant family. Given the limited numbers of such enzymes currently identified, examination of a wider range of species is required to determine the extent of their phylogenetic ranges and origins. Nevertheless, some structure–function relationships studies have been carried out, providing access to the full array of alternatively coupled products.^18–20^ The derivation of such activity from the CPT family hints at the potential of such enzymes to also catalyze class I terpene synthase reactions – *i.e.*, much as has been observed with the IDSs as these catalyze functionally analogous reactions that differ only in product configuration (*c.f.*, Fig. 1 and Scheme 1). Of particular relevance here, there are irregular diterpenoids and, although the relevant enzymes have not been identified, it has been suggested at least one of these might mediate (*via* cyclobutylation) a much more complex cyclization reaction (Fig. 2B).^21^

## 2 Terpene synthases

Terpene synthases form the initial hydrocarbon backbones that define the various families/types of (di)terpenoids,^13^ and have been termed metabolic gatekeepers in the evolution of such biosynthesis.^23^ As noted above class I terpene synthase activity only requires the helical-bundle α domain and those found in microbes are simply composed of this single domain. By contrast, those commonly found in plants (*i.e.*, the TPSs) contain at least one additional domain. Nevertheless, microbial-like terpene synthases composed of just an α domain whose sequence phylogenetically groups with those from microbes rather than the plant TPSs are found in nonseed plants (representing early diverging lineages). Notably, these are involved in mono- and sesqui-, but not di-terpene biosynthesis.^24^ Accordingly, these enzymes will not be further discussed here. However, this functional split, with smaller (mono- and sesqui-) terpenes produced by a separate enzymatic family in nonseed plants, highlights the use of TPSs for at least diterpenoid biosynthesis throughout all land plants, suggesting the ancestral TPS served such a function. Indeed, given the use of *ent*-kaurene derived diterpenoids as phytohormones in all extant land plants it has been hypothesized the evolution of diterpenoid metabolism in plants was initiated by acquisition of a bifunctional cyclase that first converts GGPP to *ent*-CPP (Fig. 1). Indeed, the fused enzyme required for this initial cyclization reaction provides the source of the additional domain(s) found in all TPSs.^13^

The ancestral TPS is presumed to have functioned as both an *ent*-CPP synthase (CPS) as well as subsequently acting *ent*-kaurene synthase (KS), with extant examples of such bifunctional CPSKSs found in some nonseed plant species. Consistent with retention of the allylic diphosphate ester bond in *ent*-CPP, the KS exhibits class I terpene synthase activity (Fig. 1). By contrast, CPS catalyzes a carbocationic bicyclization reaction initiated by protonation of the terminal olefin of GGPP, forming an *ent*-labda-13-en-8-yl^+^-15-diphosphate that is immediately deprotonated at the methyl adjacent to the carbocation.

**Scheme 2:**
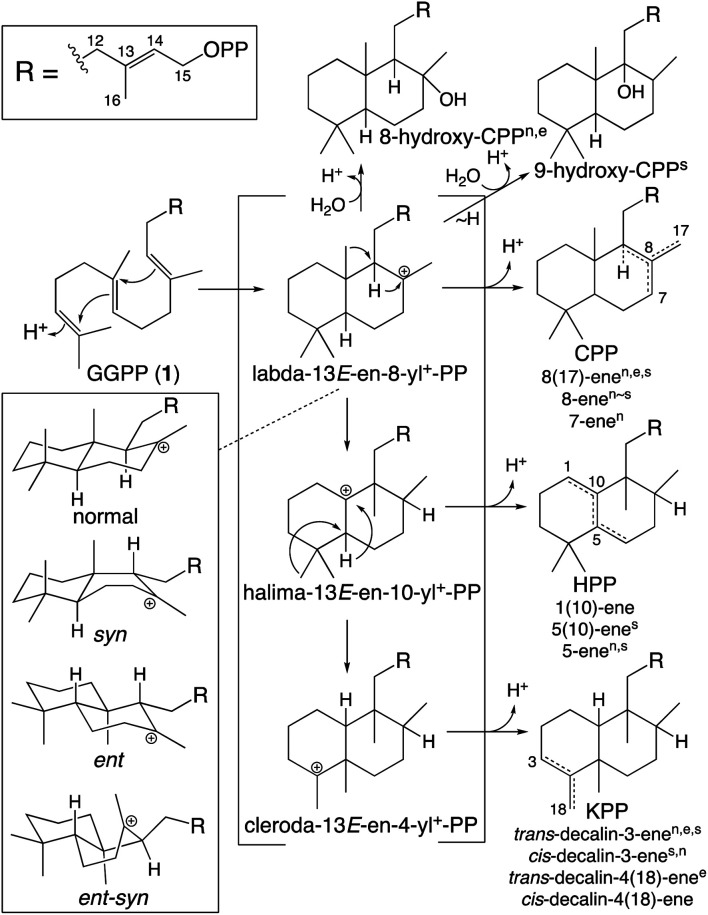
Basic DTC catalysis, including initial bicyclization of the decalin core and subsequent rearrangement (PP = diphosphate). Also shown are known stereoisomers for the initial decalin bicycle, with derived products from identified DTCs indicated by superscript (^n^normal, ^e^*ent*, ^s^*syn*, no *ent-syn* have yet been identified; note that KPP, kovalenyl diphosphate, is used to distinguish this from the labdane, CPP).^25^ In addition, rearrangement of the initially formed decalin bicycle also occurs, with one such (fungal) DTC identified.^26^

While falling into the TPS family, CPS provides an example of the functionally distinct class II diterpene cyclases (DTCs). These prototypically produce a labda-13-en-8-yl^+^-15-diphosphate carbocation intermediate. Thus, their activity has been used to define the labdane-related diterpenoid super-family.^6^ However, it must be noted that DTCs can yield rearranged products and the vast majority of this super-family are no longer labdanes (*e.g.*, even those derived from CPP, due to further cyclization catalyzed by class I diterpene synthases such as KS). In addition, as indicated by the *enantiomeric* (*ent*-) nomenclature relevant to phytohormone biosynthesis, the initial bicyclization of GGPP yields one of four distinct stereoisomers of labda-13-en-8-yl^+^-15-diphosphate. Altogether DTCs can form close to 100 different compounds, although those identified to-date only yield 20 distinct products (Scheme 2).

**Fig. 3:**
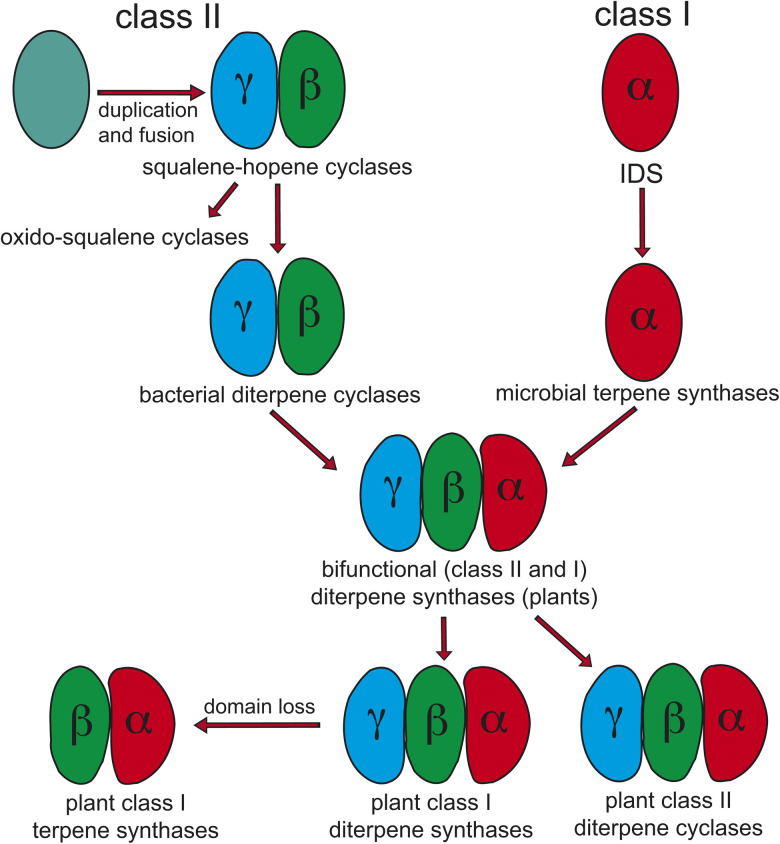
Current view of the evolutionary origin of plant terpene synthases (adapted from ref. 13).

Notably, protonation-initiated carbocationic cyclization reactions also are observed in triterpenoid biosynthesis, as catalyzed by oxido-squalene cyclases (OSCs) and squalene-hopene cyclases (SHCs), which share structural homology with the DTCs, leading to their common grouping as class II terpene cyclases. In particular, catalysis is carried out in an active site situated between a pair of (αα)_6_-barrel domains, which have been hypothesized to share a common origin – *i.e.*, *via* gene duplication and fusion.^12^ Consistent with this hypothesis it has recently been shown that a single such domain is capable of catalyzing an analogous protonation-initiated cyclization reaction with a geranylgeranyl dihydroxybenzoate derivative.^27^ The more typical pair of domains have been termed β and γ, with a primary sequence order of γβ.^13^ Fusion of a di-domain DTC, as still found in bacteria, to create the ancestral CPSKS provides the origin of the additional domains found in the TPS family (Fig. 3). The relevant domains are found in the primary sequence order γβα, with an extensive interface observed between the β and α domains.^13^ This appears to be the basis for the presence of βα di-domain architecture throughout the TPS family, wherein the γ domain has been lost several times following loss of class II (DTC) activity.^28^ A particularly early example of such domain loss seems to have occurred prior to establishment of seed plants (spermatophytes), leading to the TSP-d1 group found in gymnosperms and, hence, TPS-a, b & g subfamilies found in angiosperms.^29^

**Scheme 3:**
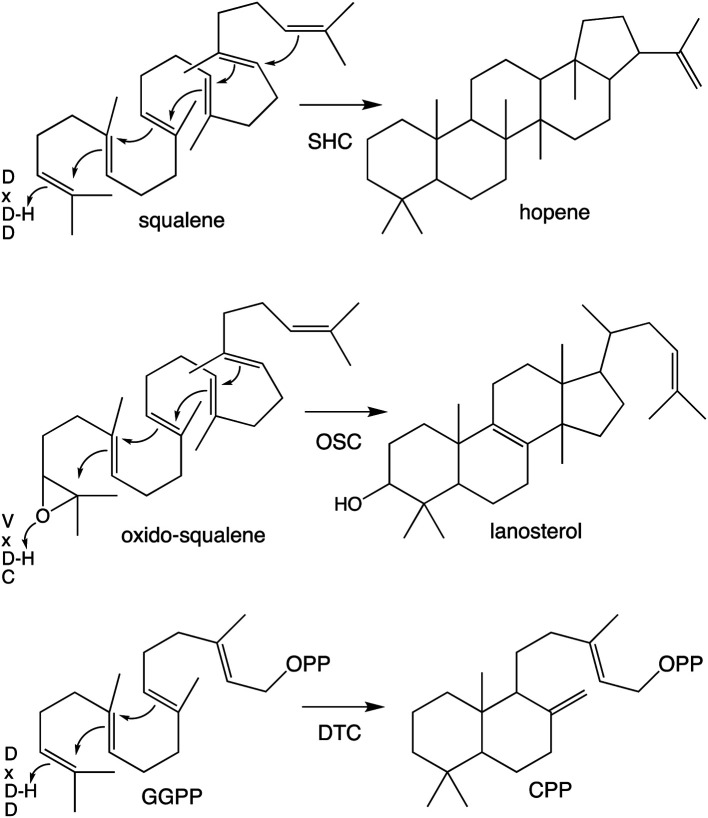
Examples of class II catalyzed cyclizations with associated catalytic acid motifs.

The homology between DTCs and triterpene cyclases (SHC and OSC) extends to use of a conserved aspartic acid residue, located in the β domain, as the catalytic acid initiating cyclization *via* protonation. In the case of the ancestral SHCs as well as DTCs this is the ‘middle’ residue of a highly conserved DxDD motif, reflecting their common need to carry out the more energetically difficult protonation of an olefin relative to the less strenuous epoxide protonation mediated by OSCs (Scheme 3). Accordingly, the presence of this motif has been used as a signature of DTC activity. Similarly, the Mg^2+^-binding motifs in class I terpene synthases (matching the pair of DDxxD motifs from the IDS family), both a highly conserved DDxxD but also less well conserved (derived) NSE/DTE motifs,^30^ serve as signatures of such activity. While loss of the γ domain is clearly indicative of monofunctional class I TPSs, these motifs are useful in elucidating basic activity of the tri-domain TPSs found in all land plants – *i.e.*, at least one such TPS is required for phytohormone biosynthesis. Before spermatophyte divergence the ancestral CPSKS underwent gene duplication and subfunctionalization to yield the separate CPSs and KSs found in all extant species. Both of these retain the ancestral γβα tri-domain architecture, albeit with some degradation of the no longer relevant domains, particularly including loss of the corresponding catalytic motifs. Moreover, the KS and/or CPS(KS) required for phytohormone biosynthesis have given rise, *via* repeated gene duplication and neofunctionalization to more specialized metabolism, to extended sub-families of TPSs – *i.e.*, CPS(KS)s to TPS-c and KSs to TPS-e − in a lineage specific manner. For example, as has been discussed for the Poaceae^31^ and Lamiaceae plant families.^32^

**Fig. 4:**
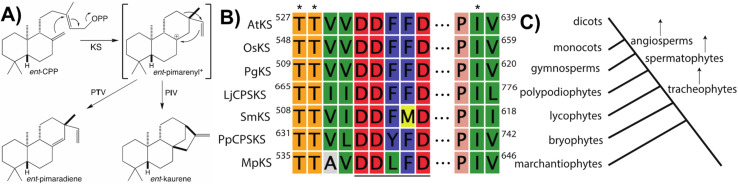
KS reaction and associated motifs. (A) Catalyzed reaction and effect of noted threonine for isoleucine substitution, with relative p*K*_a_ for carbocations and hydroxyls shown. (B) Conservation of DDxxD (underlined) characteristic of class I TPSs, with extension and PIV motif (with key residues indicated by asterisks) specific to the KSs involved in phytohormone biosynthesis across all land plants shown here by alignment of representative examples spanning land plant evolution (AtKS, *Arabidopsis thaliana*; OsKS, *Oryza sativa*; PgKS, *Pinus glauca*; LjCPSKS, *Lygodium japonicum*; SmKS, *Selaginella moellendorffii*; PpCPSKS, *Physcomitrella patens*; MpKS, *Marchantia polymorpha*). (C) Approximate phylogenetic tree corresponding to sequences shown in panel B.

Notably, beyond the catalytic motifs associated with class I or II activity, signature motifs for the KSs and/or CPS(KS)s involved in phytohormone biosynthesis have been recently defined. In the case of the KSs it had been previously proposed that a particular isoleucine was important for production of *ent*-kaurene, with substitution of threonine leading to production of an isopimaradiene instead, representing premature deprotonation of the usual carbocation cascade reaction. However, this hypothesis had only been tested in KSs from spermatophytes,^33,34^ but has now been demonstrated to hold true for KSs across the full phylogenetic range of land plants,^35^ where it is conserved within a KS-specific PIx motif (Fig. 4). Moreover, while the altered side-chain was originally proposed to simply stabilize the corresponding pimarenyl carbocation intermediate,^36^ it has since been recognized that unactivated hydroxyl groups are sufficient to act as a general base for carbocation deprotonation (p*K*_a_ for protonated alcohols is −1 to −4 while for unconjugated carbocations is less than −10).^37^ This result not only highlights the requirement for an otherwise inert active site, but also suggests how enzymatic engineering might be accomplished. In addition, it has been recently recognized that the KSs involved in phytohormone biosynthesis contain a similarly KS-specific extension of the DDxxD motif to TTxxDDxxD (Fig. 4), although the functional role of this pair of threonines remains unknown.^38^ Insight into this may be provided by structural analysis, which is currently lacking for any KS or, indeed, any member of the TPS-e subfamily.

**Fig. 5:**
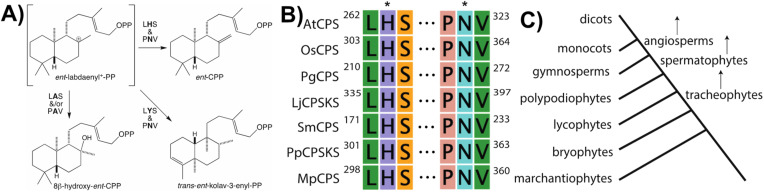
CPS reaction and associated motifs. (A) Catalyzed reaction and effect of noted substitutions. (B) Conservation of motifs (with key residues indicated by asterisks) specific to the CPSs involved in phytohormone biosynthesis across all land plants shown here by alignment of representative examples spanning land plant evolution (AtCPS, *Arabidopsis thaliana*; OsCPS, *Oryza sativa*; PgCPS, *Pinus glauca*; LjCPSKS, *Lygodium japonicum*; SmCPS, *Selaginella moellendorffii*; PpCPSKS, *Physcomitrella patens*; MpCPS, *Marchantia polymorpha*). (C) Approximate phylogenetic tree corresponding to sequences shown in panel B.

With regards to CPSs, given conservation of the DxDD motif as the general acid catalyzing initiating protonation of the terminal olefin of GGPP, it was difficult to discern the functional group serving as the catalytic base, whose position may then vary with differential product outcome. However, based on structural analysis of the CPS from *Arabidopsis thaliana* (AtCPS),^39,40^ it has now been determined the CPSs specifically involved in phytohormone biosynthesis contain a histidine and asparagine dyad, located in the γ domain across the active site cleft from the DxDD motif, which help tightly coordinate a water molecule likely serving as the catalytic base.^41^ These two residues appear to be functionally conserved across the full phylogenetic range of land plants, with alanine substitution invariably leading to an alternative hydroxylated product derived from the addition of water to *ent*-labda-13-en-8-yl^+^-15-diphosphate (Fig. 5).^42^ Moreover, the identity of the residues at these positions and/or those immediately neighboring has been demonstrated to be important for controlling product outcome even more broadly.^43–48^ These then define two distinct LHS and PNV motifs, which are specific to those CPSs involved in phytohormone biosynthesis, as the production of *ent*-CPP can be accomplished with alternative catalytic bases.^42^

Notably, the observed conservation patterns suggest these motifs should be predictive for KS and/or CPS(KS) activity. Indeed, this hypothesis has recently been explicitly tested *via* examination of TPSs family members from a wide array of early diverging nonseed plant species, where these motifs were found to correlate with at least the underlying production of *ent*-kaurene and/or *ent*-CPP, respectively (Table 1).^29^ In addition, this study helped clarify the early gene duplication and sub/neo-functionalization events that underlie evolution of the TPS family, indicating two such events occurred before and/or during the early divergence of land plants. The clearest involved neofunctionalization, leading to a bifunctional enzyme that no longer produced *ent*-kaurene and so was diverted to more specialized metabolism, serving as the ancestor to all TPS subfamilies other than TPS-c and TPS-e. The other involved subfunctionalization, leading to a monofunctional KS, which gave rise to the TPS-e subfamily, with members found in all major plant lineages other than mosses (bryophytes) and ferns (polypodiophytes). The absence of this subfamily in these two disparate plant lineages is hypothesized to stem from retention of the bifunctional CPSKS at least until the divergence of ferns, consistent with the presence of such enzymes in extant nonseed species including ferns. Given the lack of channeling between the class II and I active sites in bifunctional enzymes,^49^ a monofunctional KS can react with the *ent*-CPP released by the remaining CPSKS, although it is unclear if this activity is sufficient to explain retention of both.

**Table 1:** Predictive power of TPS motifs^a^

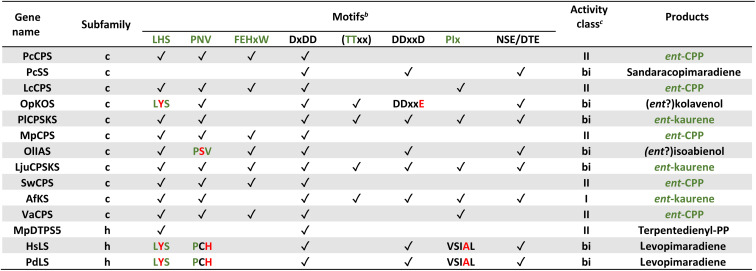

a Modified (selected examples) from ref. 29.

b For all motifs “✓” indicates conservation, while red text indicates important changes discussed in text.

c “I”, “II” and “bi” depict class I, class II and class I/class II bifunctional TPS, respectively, with green text indicating expected production of *ent*-CPP and/or *ent*-kaurene (CPS and/or KS activity, respectively) for phytohormone biosynthesis.

**Fig. 6:**
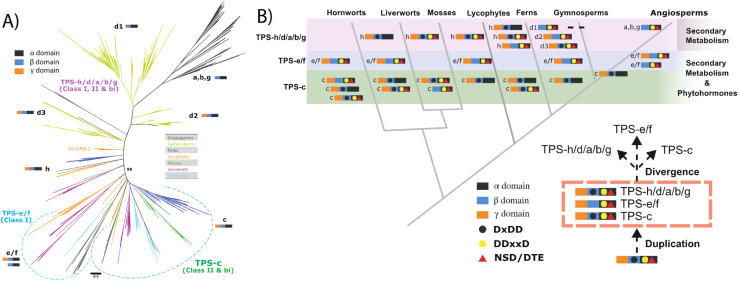
TPS evolution (derived from ref. 29). (A) Unrooted phylogenetic tree for the TPS family. (B) Model of TPS evolution depicting phylogenetic relationship of land plants, indicated by grey lines, with TPS domain structure and catalytic motifs as defined in text and indicated in legend.

Increased resolution of early events in TPS evolution also clarified key events in the remainder of this family – *i.e.*, the other subfamilies (a, b, d, g & h; note TPS-f is composed of a few TPS-e derivatives no longer acting in labdane-related diterpenoid biosynthesis). Given their dedication to more specialized metabolism and apparently monophyletic origin, these are termed here clan S. In addition, this recent analysis reveals deep divisions within the TPS-d and TPS-h subfamilies. While those from TPS-h largely follow plant lineage, those from TPS-d (by definition limited to gymnosperms) are informative. In particular, while the previously defined TPS-d3 group largely consists of bifunctional diterpene cyclases (much like the TPS-h subfamily), it is now clear this gave rise to the TPS-d2 group, characterized by monofunctional class I mono- or sesqui-terpene synthase activity yet retaining the ancestral γβα tri-domain architecture, this then gave rise to the TPS-d1 group wherein the now defunct γ domain is lost, which in turn gave rise to all the remaining subfamilies found in angiosperms (Fig. 6).^29^

**Scheme 4:**
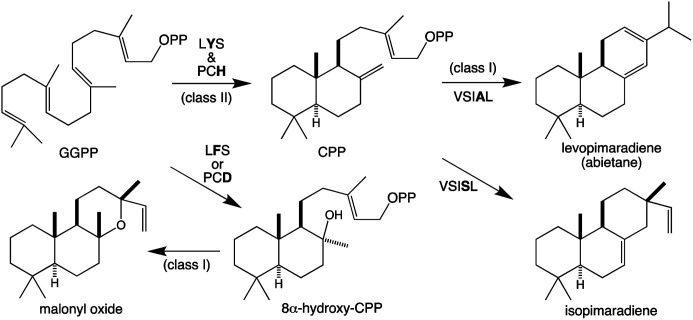
Reactions catalyzed by bifunctional TPS-d3 group members with key motifs and effects of noted substitutions, as well as efficiently catalyzed heterocyclization.

Similar to KS and/or CPS/KS, previous work defined key motifs for the production of normal CPP and subsequent conversion to an abietane catalyzed by the majority of the known TPS-d3 group members, which provided further insight into the evolution of such activity (Scheme 4). In the TPS-d3 class II active site the LHS and PNV motifs defining the catalytic base in the ancestral CPS(KS)s involved in phytohormone biosynthesis have been converted to LYS and PCH. The side chains of the highlighted tyrosine and histidine are directly hydrogen-bonded together, and seem to cooperatively act as the catalytic base.^44^ Similar to CPSs, certain substitutions for these two residues lead to the incorporation of water – *i.e.*, production of 8α-hydroxy-CPP.^43,44^ Notably, the ability of the class I active site in these enzymes to further react with this alternative product led to the realization that such enzymes, including KSs, can efficiently catalyze heterocyclization with the stereochemically appropriate hydroxylated substrate.^44^ In the class I active site of TPS-d3 group members, a key alanine plays an equivalent role to the key isoleucine found in the ancestral KSs. In particular, similar to the discovery of the key isoleucine in KSs,^33^ this alanine was identified by comparison of closely related abietane *versus* pimaradiene producing enzymes, with substitution by serine leading to such alternative (isopimaradiene) product outcome in abietane synthases.^50,51^ This alanine along with the surrounding residues defines a VSIAL motif highly conserved in these enzymes and is just four residues upstream of the key isoleucine in KSs. While no structures have been reported for KSs or any other members of the TPS-e subfamily (often referred to KS-like, KSLs), structural analysis of the abietane synthase from the gymnosperm *Abies grandis* (AgAS) demonstrates that the key alanine sits at a widely conserved (G1/2) helix-break.^52^ The key isoleucine in KSs then sits lower in the active site, consistent with the distinct (*enantiomeric*) stereochemical configuration of the relevant substrates. Regardless, it is notable that the two TPS-h subfamily members from lycophytes recently shown to produce abietanes (HsLS and PdLS) contained all three of these abietane synthase specific motifs and are relatively closely related to TPS-d3, suggesting that such activity may have evolved prior to the divergence between lycophytes and gymnosperms (Fig. 6 and Table 1).^29^

Even beyond conservation of these varying key motifs, the effect of certain changes within at least the class II catalytic base dyad has been hypothesized to provide additional predictive power. For example, the ancestral LHS and PNV motifs and their role in helping to coordinating a water (along with two other hydrogen bonds to the protein backbone) that serves as the catalytic base was uncovered not only by structural analysis of AtCPS,^39,40^ but the subsequent finding that alanine substitution for either the histidine or asparagine led to production of a hydroxylated derivative (8β-hydroxy-*ent*-labda-13-en-15-yl diphosphate) stemming from addition of water (Fig. 4).^41^ This effect was later demonstrated for CPSs involved in phytohormone biosynthesis from across the full phylogenetic range of land plants, with the additional finding that substitution of serine for the ancestral asparagine, as found in a natural example, also leads to such alternative product outcome.^42^ Indeed, the recent screen of TPSs from early diverging land plants found another potential example of such substitution resulting in production of this hydroxylated derivative (OlIAS; Table 1).^29^ Similarly, it was found with AtCPS that substitution of tyrosine or phenylalanine for the ancestral histidine led to a completely rearranged product – *i.e.*, *trans*-(decalin)-*ent*-kolav-3-enyl diphosphate (Fig. 5A)^48^ – which presaged discovery of two natural examples of such enzymes,^46,47^ with another potential example again found among the screen of TPSs from early diverging land plants (OpKOS; Table 1).^29^

Given the ease with which DTCs are converted to alternative product outcomes, how readily these can be further elaborated upon is a key aspect of metabolic evolution. Notably, at least some substrate promiscuity has been shown with the subsequently acting class I TPSs – *e.g.*, in KSLs from rice (*Oryza sativa*) and wheat (*Triticum aestivum*),^53,54^ as well as the Lamiaceae plant family,^55^ and even more generally.^25,56,57^ Accordingly, it seems likely changes in DTC product outcome may be immediately accommodated by subsequently acting extant enzymes, providing a means for facile metabolic evolution of labdane-related diterpenoids.

**Scheme 5:**
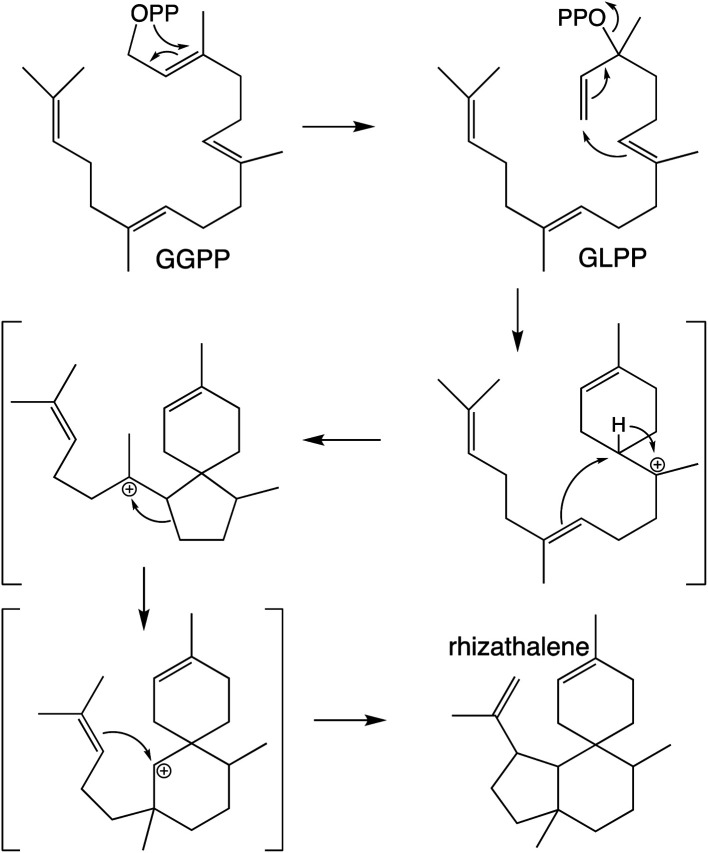
Class I TPS catalyzed isomerization and cyclization of GGPP to rhizathalene.^3^

It should be noted that the reactivity of class I terpene synthases with DTC products (*i.e.*, labdane-related diterpenoid biosynthesis) is not confined to just the TPS-e subfamily and TPS-d3 group, as such functionality has been shown for TPS-b subfamily members, at least in the *Tripterygium* genus.^58–60^ Conversely, beyond the noted derivation of TPS-e subfamily members to react directly with GGPP that was used to define the TPS-f subfamily, similar reactivity also is evident within TPS-d3 given the production of taxadiene by members of this group.^61^ The increased number of carbon–carbon double bonds in GGPP (and NNPP) relative to the precursors for smaller terpenoids (*e.g.*, farnesyl diphosphate), enables a dizzying array of routes for the catalyzed carbocation cascades, which is even further expanded upon by the ability to catalyze isomerization to the tertiary intermediate geranyllinalyl diphosphate (GLPP), enabling 1,6-cyclization (*e.g.*, Scheme 5).^1^ Thus, beyond noting that such activity was apparently re-evolved from mono- and sesqui- TPSs in angiosperms, the sheer complexity of the catalyzed reactions has stymied our understanding of such class I diterpene synthase evolution, which will not be further discussed here.

## 3 Cytochromes P450 (CYPs)

**Fig. 7:**
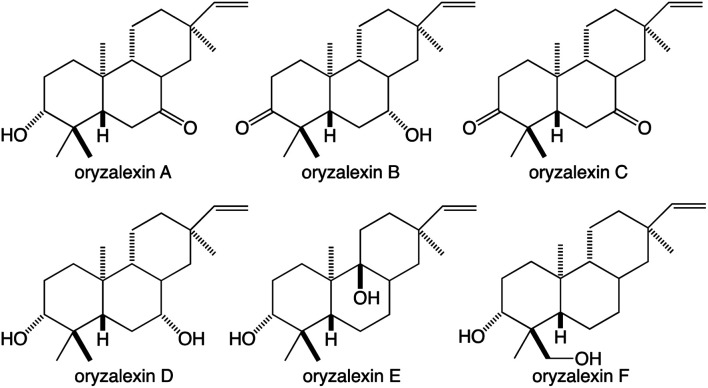
Rice oryzalexins (antimicrobial phytoalexins).

While the TPSs can generate oxygenated products *via* the incorporation of water into their carbocationic cascade reactions, these more typically construct pure hydrocarbons whose hydrophobicity requires the addition of oxygen to increase solubility and provide hydrogen bonding potential to impart specific biological activities.^1^ More specifically, given the size of their hydrocarbon chain, diterpenes are highly hydrophobic, with partition coefficients (log *P*) > 8, requiring the addition of at least two spatially separated oxy groups to acquire reasonable solubility (log *P* ≤ 5), matching the usual composition of the simplest diterpenoids with known biological activity – *e.g.*, rice oryzalexins (Fig. 7).^62^ The relevant oxygenases are almost invariably from the cytochrome P450 (CYP) super-family, whose membrane localization provides access to these hydrophobic diterpenes, particularly given the apparent exchange of such metabolites between the plastid and endoplasmic reticulum where CYPs are located (possibly *via* hemi-fusion of the outer leaflet of the outer membrane of each of these organelles).^63^ Regardless of how access is achieved, the CYPs also make essential contributions to (di)terpenoid biosynthesis.^64,65^ Phylogenetic relationships within the CYP super-family can be inferred to some extent in the associated nomenclature as, by definition, the numbered families share >40% and lettered subfamilies >55% amino acid sequence identity, with individual members assigned numbers.^66^ As an example of such nomenclature the *ent*-kaurene oxidase involved in phytohormone biosynthesis from *A. thaliana* is CYP701A3.^67^ However, given the increasing amounts of sequence data some merging of (sub)families has inevitably occurred, which has been addressed with assignment of tribes to describe such latter realized phylogenetic relationships.^68,69^

Unlike TPSs, there is very little direct derivation of CYPs from those involved in phytohormone biosynthesis to more specialized diterpenoid metabolism. Indeed, the only known examples stem from the CYP701 family, where almost all characterized members act as *ent*-kaurene oxidases, which seems to be necessary for phytohormone biosynthesis throughout all land plants,^1^ with only individual members from rice and maize found to exhibit alternative activity.^70–72^ Nevertheless, it should be noted with the subsequently acting CYP88 family (at least for gibberellin phytohormone biosynthesis occurring in vascular plants), where almost all characterized members act as *ent*-kaurenoic acid oxidases, it has been reported that a few members operate in more specialized triterpenoid biosynthesis,^73^ hinting at the possibility that others may be involved in more specialized diterpenoid metabolism. Regardless, more specialized diterpenoid biosynthesis seems to largely rely on use of the same (sub)families operating in other such metabolism. For example, members of the expansive CYP71D subfamily operate in not only diterpenoid but other types of terpenoid, and even flavonoid, biosynthesis.^74^ Nevertheless, there is some correlation between CYP (sub)family and metabolic function, at least within the various plant lineages.

**Fig. 8:**
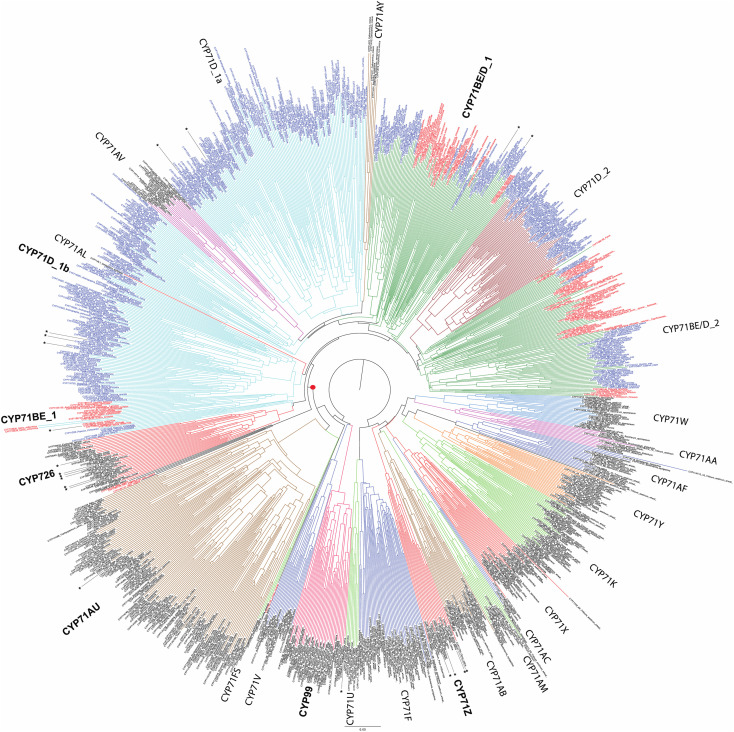
Phylogenetic tree for 21 (of 137) CYP71 subfamilies (as well as the related/subsumed CYP99 and CYP726), with members known to function in diterpenoid biosynthesis indicated by asterisks (*). Tribe described here indicated by red dot (•). Assembled using CLUSTAL Omega (neighbor-joining) from 1073 sequences selected to reduce redundancy. Note the intermixing of CYP71D and CYP71BE subfamilies arising from increased sequence coverage since their original designations.

While relatively few CYPs have been associated with diterpenoid metabolism in non-seed plants, the role of the CYP720B subfamily in conifer resin acid biosynthesis,^75^ as well as CYP725A subfamily in Taxol biosynthesis,^76,77^ has long been appreciated.^74^ Also apparent at the time of the last review^1^ were roles in diterpenoid biosynthesis played by members of the CYP71Z, CYP76M, CYP99A and CYP701A subfamilies in rice,^78^ CYP76AH subfamily in the Lamiaceae,^79,80^ and CYP71D subfamily in tobacco.^81^ Since that time, it has been shown that members of the CYP71Z,^7,8,82,83^ as well as CYP701A,^71^ subfamilies operate in more specialized diterpenoid biosynthesis throughout the Poaceae plant family.^31^ Some evidence also has been provided suggesting broader use of the CYP76M subfamily, along with possibly the CYP76L subfamily, in Poaceae diterpenoid biosynthesis as well.^84,85^ Both of these CYP76 subfamilies, as well as the CYP71Z and CYP99A subfamilies, are unique to this plant family, suggesting these may have evolved for such metabolic roles. However, rather than a common origin the CYP701A subfamily members operating in more specialized diterpenoid metabolism in rice and maize appear to have independently diverged from the ancestral role in phytohormone biosynthesis, providing an example of parallel evolution. In Lamiaceae, since the time of the last review additional roles for CYP76AH,^86^ and roles for the CYP71AU,^87^ CYP71BE,^88^ CYP71D,^89^ CYP76AK^90^ and CYP76BK^91^ subfamilies in diterpenoid biosynthesis have been reported. Again, given that the CYP76AH, CYP76BK and CYP76AK subfamilies are unique to this plant family, these may have evolved for this purpose. By contrast, the CYP71AU and CYP71BE subfamilies are found throughout eudicots, with very few characterized members, so any broader roles in diterpenoid biosynthesis are uncertain. In addition, roles for the CYP71D as well as CYP726A subfamilies have been reported in macrocyclic diterpenoid biosynthesis in the Euphorbiaceae plant family.^92–95^ The CYP71D subfamily also has been found to operate in tomato diterpenoid biosynthesis.^9^ Notably, it is now evident that the CYP71D subfamily is intermixed with the CYP71BE subfamily, and also encompasses the CYP726 family along with CYP71AV, CYP71AU and CYP71AY subfamilies as distinct clades (albeit the last two contain a few CYP71D and CYP81BE subfamily members). This tribe is fairly closely related to the CYP71Z subfamily and CYP99 family (which is a distinct clade within the CYP71 family), hinting at the possibility that these evolved for roles in (di)terpenoid biosynthesis within angiosperms (Fig. 8). However, given the range of metabolic roles played just by CYP71D subfamily members, and relative paucity of characterized members from this tribe, it should be noted that this may simply reflect parallel evolution within the highly expanded CYP71 family.

**Fig. 9:**
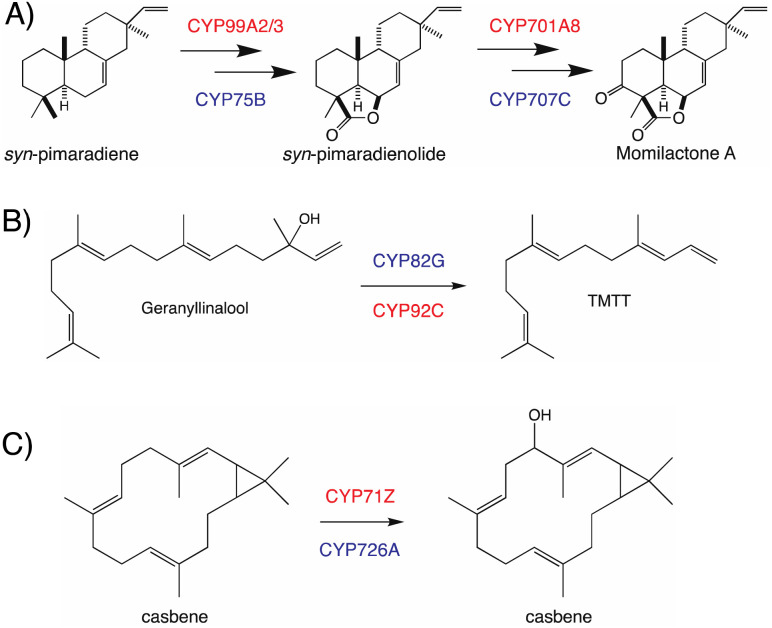
Analogous reactions catalyzed by members of distinct CYP families in different plants. (A) Momilactone biosynthesis in rice and moss, with the relevant CYPs shown in red or blue text, respectively. (B) TMTT biosynthesis in monocots and eudicots, with the relevant CYPs shown in red or blue text, respectively. (C) Hydroxylation of casbene in rice and Euphorbiaceae, with the relevant CYPs shown in red or blue text, respectively.

The use of distinct CYP subfamilies for the same purpose in diterpenoid biosynthesis in long-separated plant lineages is emphasized by comparison of momilactone production in rice *versus* the bryophyte *Calohypnum plumiforme*, with equivalent reactions catalyzed by distinct CYPs – *i.e.*, CYP99A2/3 and CYP701A8 *versus* CYP75B and CYP707C, respectively (Fig. 9A).^96^ Similarly, the cleavage of geranyllinalool to form 4,8,12-trimethyl-trideca-1,2,7,11-tetraene (TMTT, a common plant leaf volatile), which is catalyzed by CYP82G in *Arabidopsis*,^97^ but CYP92C in maize (*Zea mays*),^98^ provides another example of parallel evolution within the CYP superfamily in distinct lineages, here potentially monocot *versus* eudicot (Fig. 9B). A third example can be found in the analogous hydroxylation and subsequent oxidation of casbene catalyzed by distinct CYP subfamilies in rice (CYP71Z)^7,8^*versus* Euphorbiaceae (CYP726A).^92,95^ Although these differ in absolute stereochemistry, that just applies to the cyclopropyl moiety, with the macrocycle actually targeted for hydroxylation otherwise exactly analogous, such that this is again suggestive of parallel evolution in monocots and eudicots (Fig. 9C). Accordingly, it seems clear incorporation of CYPs into more specialized diterpenoid metabolism occurred in a lineage specific fashion, which may have some import when considering what CYP (sub)families might be involved in such biosynthetic pathways.

In addition to biosynthetic roles CYPs also serve in regulatory catabolism. Specifically for gibberellin phytohormones, as members of the CYP714 family catalyze inactivating hydroxylation reactions.^99–101^ More recently, it has been shown that members of the CYP72A subfamily also can serve an analogous role, at least in *A. thaliana*.^102^ However, members of this widespread subfamily also operate in more-specialized monoterpene indole alkaloid and triterpenoid biosynthesis,^74^ hinting that some may also play a role in such diterpenoid biosynthesis as well.

**Scheme 6:**
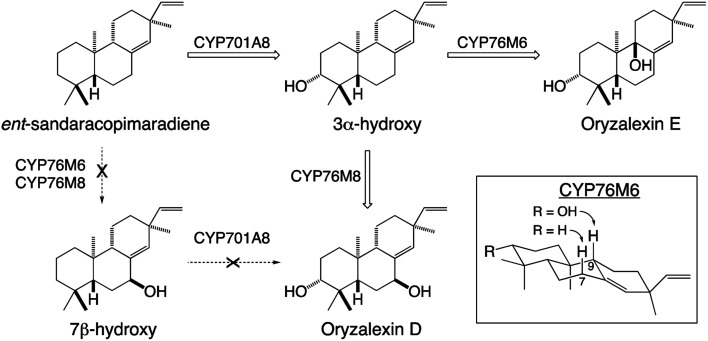
Alternative hydroxylation of *ent*-sandaracopimaradiene catalyzed by distinct members of the CYP76M sub-family from rice and their relevance to oryzalexin biosynthesis.

Given their well-known role in xenobiotic metabolism, it is perhaps not surprising that CYPs involved in diterpenoid metabolism exhibit a substantial degree of promiscuity. This includes the ability to react with metabolites at numerous stages in a variety of biosynthetic pathways, demonstrating the ability to act on not only distinct hydrocarbon backbones but also with variable modifications – *e.g.*, in rice CYP701A8 catalyzes hydroxylation at carbon 3 (C3) as an early step in phytocassane and oryzalexin biosynthesis,^70^ but as a later step in momilactone biosynthesis,^84,103^ although it also can react (albeit less efficiently) at an earlier step as well.^72^ The latter type of promiscuity has been found with many CYPs and indicates that the relevant biosynthetic processes may operate as metabolic grids rather than linear pathways. By contrast, in certain cases it has been found that previous modification can block activity. For example, in rice oryzalexin biosynthesis CYP701A8 catalyzes C3α-hydroxylation of *ent*-sandaracopimaradiene,^70^ but does not act on the 7β-hydroxy derivative.^62^ In other cases, previous modification alters regiospecificity – *e.g.*, CYP76M6 and CYP76M8 both catalyze 7β-hydroxylation of *ent*-sandaracopimaradiene, but only CYP76M8 does so with the C3α-hydroxy derivative, forming oryzalexin D, while CYP76M6 instead catalyzes C9-hydroxylation, forming oryzalexin E (Scheme 6).^62^ On the other hand, the former type of promiscuity indicates that certain CYPs may operate in a range of biosynthetic processes – *e.g.*, CYP701A8 in phytocassane, oryzalexin and momilactone biosynthesis (*c.f.*, Fig. 9A and Scheme 6). In other cases, such promiscuity leads to apparent redundancy – *e.g.*, in rice the closely related CYP76M7 and 8 both can catalyze C11 hydroxylation for phytocassane biosynthesis and C6 hydroxylation for momilactone production.^72,104,105^ However, these appear to be differentially regulated such that they hold primary metabolic roles, with CYP76M7 more important for phytocassane biosynthesis while CYP76M8 is more important for momilactone biosynthesis.^84^ Even beyond their ability to act on native metabolites, a number of CYPs from diterpenoid metabolism have been found to also react with non-native diterpenes. For example, the CYP701A3 required for phytohormone biosynthesis in *A. thaliana* has been shown to act on a wide range of diterpenes beyond the native substrate *ent*-kaurene, none of which are found in this plant species.^106^ An arguably more interesting example can be found with CYP76M8, which has been found to react with labdane-related diterpene stereoisomers not found in rice, but are present in other Poaeceae species such as wheat.^104^ Such CYP promiscuity further highlights the potential for facile metabolic evolution in diterpenoid biosynthesis and even beyond.

**Scheme 7:**
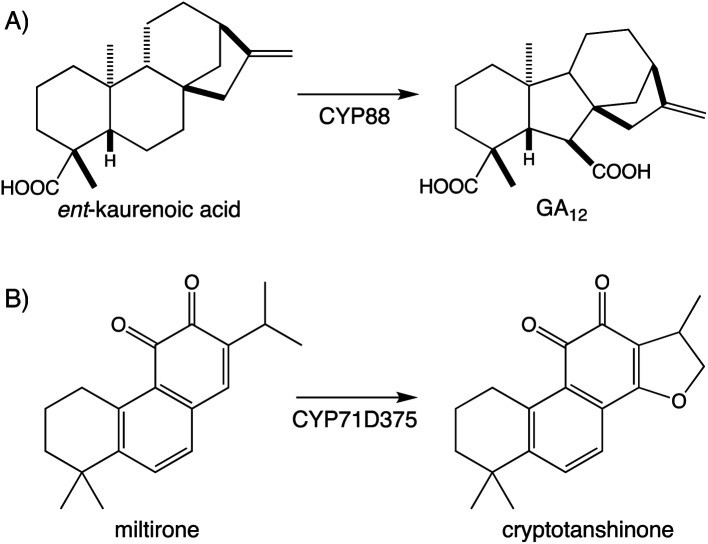
Examples of CYP catalyzed rearrangement and heterocyclization in diterpenoid biosynthesis. (A) Rearrangement in gibberellin (GA) biosynthesis. (B) Cyclic either formation (heterocyclization) in tanshinone biosynthesis.

Beyond the prototypical hydroxylation CYPs can catalyze more complex reactions.^107,108^ These include formation of heterocycles as well as modification of the hydrocarbon backbone, which often define particular families of diterpenoid natural products. A particularly widespread example can be found with the ring contraction catalyzed by CYP88A subfamily members, which transforms the kaurane 6-6-6-5 ring system to the 6-5-6-5 ring system characterizing the gibberellins that serve as phytohormones in vascular plants, which has long been recognized.^109^ Since the last review,^1^ it has been shown that CYP71D subfamily members form the epoxy ring characterizing tanshinones from the Chinese medicinal herb Danshen (*Salvia miltiorrhiza*),^89^ and that CYP99A & CYP76M subfamily members cooperatively form a hemi-aldehyde as a precursor to the lactone ring characterizing the rice momilactones.^84,103^ These examples emphasize the importance of CYP activity to diterpenoid biosynthesis (Fig. 9A and Scheme 7).

While there are relatively few structure–function studies of plant CYPs, some work has been reported with those involved in diterpenoid biosynthesis. In particular, this has most recently been focused on those from the Lamiaceae CYP76AH subfamily, where the originally characterized CYP76AH1 catalyzes C12-hydroxylation of abietatriene to form ferruginol, representing the base structure for the phenolic abietane diterpenoids commonly found in this plant family.^79,80^ Notably, later characterized subfamily members were found to act as multifunctional oxidases, catalyzing C11-hydroxylation and formation of a C7-keto group as well.^110^ It was first found that three mutations were sufficient to impart C11-hydroxylase activity to CYP76AH1.^111^ Moreover, building on the X-ray crystal structure reported for CYP76AH1,^112^ it was further reported that use of just two of these mutations enabled full multifunctional oxidase activity.^113^ It seems likely that additional enzymatic structure–function studies will be reported in not only this, but other CYP subfamilies involved in diterpenoid biosynthesis in the near future.

## 4 Downstream tailoring/decorating enzymes

**Scheme 8:**
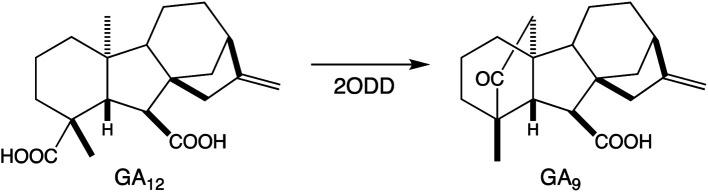
2ODD catalyzed (complex) reaction in gibberellin (GA) biosynthesis.

Relatively little is known about the subsequently acting tailoring enzymes involved in (di)terpenoid biosynthesis. These are generally soluble enzymes that act on the now soluble metabolites produced by TPSs and CYPs, but can continue to add oxygen to increase polarity and hydrogen-bonding capacity. For example, the final steps in gibberellin phytohormone biosynthesis, as well as several regulatory/catabolic reactions, are catalyzed by 2-oxoglutarate-dependent dioxygenases (2ODDs).^109^ These form a large family in plants, particularly *via* expansion of the DOXC class that includes the gibberellin oxidases.^114^ More recently, roles for such DOXC-2ODDs in tanshinone biosynthesis have been reported,^115,116^ demonstrating that members of this enzymatic family operate in more specialized diterpenoid metabolism as well. Similar to the CYPs, 2ODDs can catalyze more than just hydroxylation, with examples including demethylation (coupled to lactone ring formation) in gibberellin biosynthesis and carbon–carbon double bond formation in tanshinone biosynthesis (Scheme 8).

**Scheme 9:**
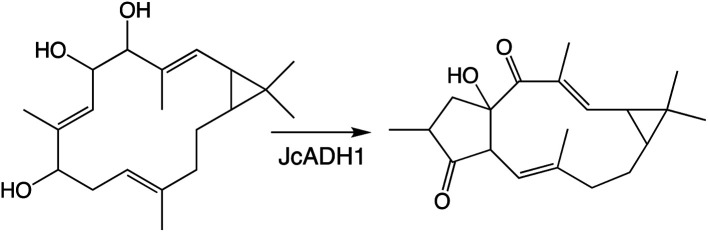
SDR catalyzed cyclization from Euphorbiaceae macrocyclic diterpenoid biosynthesis.

Another enzymatic super-family whose activity can lead to the addition of oxygen is the short-chain alcohol reductases/dehydrogenase (SDRs), albeit only *via* oxidation of aldehydes in their *gem*-diol form to a carboxylic acid, some families of which have been specifically expanded in plants.^117^ Roles for SDRs in more specialized diterpenoid metabolism has been reported since the last review, which did not cover such enzymes.^1^ In particular, SDR activity relevant to biosynthesis of the rice oryzalides^118^ and momilactones,^84,103^ as well as macrocyclic diterpenoids in Euphorbiaceae.^93,119^ Notably, in the Euphorbiaceae one of the identified SDRs catalyzes rearrangement (further cyclization) of the hydrocarbon backbone (Scheme 9),^93^ while another partitions metabolites between two distinct pathways,^119^ emphasizing the important role these also can play in (di)terpenoid biosynthesis.

Regardless of origin, the presence of hydroxy groups provides a facile target for addition of larger functional groups. Only a few such transferases are known in diterpenoid metabolism. Early work identified several types of transferases involved in Taxol biosynthesis.^76,77^ Several UDP-dependent glycosyltransferases have been identified from stevioside biosynthesis as well.^120,121^ More recently, two aceyltransferases have been identified for forskolin biosynthesis in *Coleus forskohlii*.^86^ In each case, these functional groups are critical for the desired biological activity, highlighting the important role these enzymes can play in diterpenoid biosynthesis.^76,77,122^

## 5 Biosynthetic gene clusters

The presence of biosynthetic gene clusters (BGCs)s and their implications for metabolic evolution within plants is a topic of some interest.^123–125^ Here the discussion will be largely limited to those associated with diterpenoid metabolism. At the time of the last review just two such BGCs were known.^1^ Both were found in rice, are involved in producing labdane-related diterpenoids and were discovered based on the early sequencing of the rice genome.^126,127^ In particular, as this not only enabled biochemical investigation of its arsenal of sequentially acting class II diterpene cyclases (CPSs) and class I synthases (KSLs),^128,129^ but also recognition of their functional clustering.^130–132^ The accompanying CYPs and SDRs were latter shown to operate on the products of the co-clustered CPS and KSL(s),^62,70,104,133–136^ meeting the formal plant BGC definition – *i.e.*, at least three genes of distinct evolutionary origin contributing to a shared metabolic pathway.^124^ Since that time another diterpenoid BGC has been identified in rice,^7,8^ and the three rice diterpenoid BGCs shown to exhibit varying degrees of conservation across the level of species^7^ and genus,^85^ as well as the Poaceae plant family more broadly.^137,138^ In addition, a diterpenoid BGC conserved within the Lamiaceae^32^ and another conserved within the Euphorbiaceae^92,94,95^ along with one from a bryophyte,^96^ also have since been reported.

**Fig. 10:**
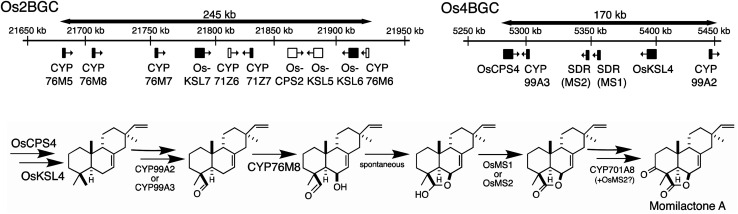
Rice biosynthetic gene clusters (BGCs) associated with labdane-related diterpenoids from chromosome 2 (Os2BGC) and 4 (Os4BGC), along with recently elucidated pathway for momilactones, requiring genes from both BGCs (largely Os4BGC, note that the positioning of MS1 and MS2 has been corrected relative to our other publications, but also CYP76M8 from Os2BGC, as well as CYP701A8 from a tandem gene array).

Arguably the best understood diterpenoid BGCs are those from rice, which were among the first to be identified and have been extensively investigated, providing a wide overview of BGC function and evolution even more generally (Fig. 10). For example, the range of genes located in the BGC on rice chromosome 2 (Os2BGC) includes multiple KSLs, which clearly lead to distinct biosynthetic pathways and are not all similarly inducible, argues against co-regulation as a driver of BGC assembly.^134^ Moreover, the first evidence that negative selection pressure against interruption of particular biosynthetic pathways might be involved in BGC assembly was provided from genetic studies with the BGC on rice chromosome 4 (Os4BGC) associated with production of the momilactones, where a knock-out line for the relevant OsKSL4 was found to exhibit reduced seed germination rates.^139^ This is coupled with the expected positive selection pressure for production of the final bioactive natural product,^140^ in the case of Os4BGC the momilactones that not only act as phytoalexins and allelochemicals,^139,141,142^ but may also play a role in regulating stomatal opening in certain genetic backgrounds (*i.e.*, at least the Kitaake cultivar).^143^ Together this supports co-inheritance as the primary driving force for assembly of at least the Os4BGC.

However, elucidation of the momilactone biosynthetic pathway further revealed that it is not entirely encoded by the Os4BGC, as genes from the Os2BGC (also involved in labdane-related diterpenoid production), as well as a separate CYP701A tandem gene array, are also required.^84^ Intriguingly, genetic dissection of these two rice BGCs further demonstrated that loss of the Os2BGC, and even more specifically the two (partially redundant) CYP76M(7/8) genes involved in momilactone biosynthesis, led to a deleterious (lesion mimic) phenotype, which was dependent on the presence of the Os4BGC, again consistent with negative consequences for interruption of momilactone biosynthesis.^105^ The lack of recruitment of the relevant gene to the Os4BGC may be due to tandem CYP76M gene duplication within the Os2BGC, which has been suggested to have an earlier origin within the *Oryza* genus.^85^ In particular, the original CYP76M subfamily member presumably originally operated in biosynthesis of the phytocassanes with which this BGC is most closely associated – *e.g.*, this contains the upstream OsCPS2 and OsKSL7 ref. 134 – but could also carry out the relevant hydroxylation reaction for momilactone biosynthesis. Although the two derived genes appear to be undergoing subfunctionalization, as CYP76M7 is more important for phytocassane biosynthesis while CYP76M8 is more important for momilactone production,^84^ there simply may not have been sufficient time and/or selective pressure for the transfer of CYP76M8 from the c2BGC to the c4BGC. A similar argument might be made for the lack of recruitment of CYP701A8, but it should be noted that its location in a tandem array of CYP701A orthologs, including the requisite kaurene oxidase for gibberellin phytohormone biosynthesis, ensures its co-inheritance. The multiple roles CYP701A8 plays in various biosynthetic processes further complicates the selective pressures on this gene.

Similar gene duplication appears to have occurred in the Os4BGC, where apparently redundant CYP99A(2 & 3) subfamily members are present (see Fig. 9A), along with at least partially redundant SDRs (both termed momilactone A synthase; MAS1 & 2).^84^ However, this is even more widespread in the Os2BGC, which actually contains four closely related CYP76M subfamily members (albeit another subfamily member that falls within this phylogenetic clade is located elsewhere in the rice genome), along with two from the CYP71Z subfamily, and three KSLs (Fig. 7). While many of these exhibit distinct biochemical activity, the two KSLs not involved in phytocassane biosynthesis (OsKSL5 & 6) catalyze identical product outcome, at least in certain genetic backgrounds (*i.e.*, the IR24 cultivar),^144^ although not others (*i.e.*, the Nipponbare cultivar),^132^ yet appear to have been recruited latter in the Os2BGC assembly process and are clearly a result of subsequent tandem gene duplication.^85^ In these latter cases, it is unclear what drove the sweep of the BGC containing the (partially) redundant duplicated genes throughout the population (or if these simply accompanied other desirable loci selected for during domestication).

The most recently identified rice diterpenoid BGC, located on chromosome 7 (Os7BGC), contains a single TPS along with at least two closely related members of the CYP71Z subfamily (see Fig. 1B).^7,8^ This composition does not meet the formal BGC definition, as the CYP71Z subfamily members appear to have arisen from local gene duplication. However, the CYP71Z subfamily members have diverged to catalyze distinct reactions in the relevant biosynthetic pathway, providing insight into the selective pressure for this gene duplication event. The Os7BGC also is noteworthy for two other features. First, in its basis from *ent*-casbene, resulting from direct reaction with GGPP by the encoded TPS, which draws an interesting parallel with the Euphorbia diterpenoid BGC, as this similarly encodes casbene synthases, and both BGCs contain CYPs carrying out analogous hydroxylation reactions as well (*i.e.*, from the CYP71Z and CYP726A subfamilies, as mentioned above). However, despite these biochemical similarities the two BGCs are not otherwise homologous (*i.e.*, the TPSs are not closely related and each BGC contains distinct CYP families), indicating their independent assembly. Consistent with the co-inheritance model, the macrocyclic casbene has been reported to exert phytotoxic membrane disruption, which is not expected to vary with the limited configurational difference between enantiomers, suggesting analogous negative selection pressure against casbene synthases in the absence of downstream acting CYPs.^8^ Second, production of the diterpenoid phytoalexin (*ent*-10-oxodepressin) encoded by the Os7BGC exhibits a limited distribution, almost exclusively found in the japonica subspecies (ssp.) of rice, with only one example found in the >10 examined lines of ssp. indica.^7^ Given the usually distinct growth conditions for these two major subspecies, it seems likely that the observed distribution of the Os7BGC might reflect a selective advantage for production of *ent*-10-oxodepressin in the usual conditions for ssp. japonica but not indica. This is consistent with recently advanced hypothesis for BGC assembly,^123^ as such concentrated genetic architectures are expected to arise when a locally adaptative trait is evolving within a wider population and there is migration between local populations. However, it should be noted that *ent*-10-oxodepressin has only been reported to act as a phytoalexin against the same fungal blast pathogen (*Magnaporthe oryzae*)^8^ and bacterial leaf blight (*Xanthomonas oryzae*)^7^ targeted by the other rice diterpenoid phytoalexins,^141,142^ whose production relies on the two other more widely distributed BGCs.

By contrast, the Os4BGC exhibits a dramatically broader distribution, with homologous BGCs found in not only other species from the *Oryza* genus that similarly produce momilactones,^85^ but also in barnyard grass (*Echinochloa crus-galli*)^145^ and wheat,^138^ as well as even more widely throughout the grasses.^146^ Given the evolutionary divergence between rice and especially barnyard grass, as these are separated between the two major clades in the Poaceae plant family, this BGC appears to provide an example of lateral gene transfer.^137,146^ However, while nothing is known about the metabolic pathway encoded by the BGC from barnyard grass, it has been shown that at least the CPS and KSL from the relevant wheat BGC exhibits divergent function.^138^ Accordingly, this BGC does not appear to have evolved for momilactone production, and not only the identity but also function of the resulting labdane-related diterpenoid remains unclear in the other genera where it is found. On the other hand, it must be noted that the bryophyte *C. plumiforme* has been found to not only produce momilactones but also contain an associated BGC, which clearly independently evolved from that found in the Poaceae despite proceeding *via* functional analogous biosynthetic transformations.^96^ Given the parallels to what has been observed in rice, potential phytotoxicity of the relevant intermediates has been suggested, which would again be consistent with a primary role for co-inheritance in driving assembly of this BGC as well.^137^

## 6 Conclusions

As described here, the origins of plant diterpenoid metabolism can be traced back not just to the ubiquitous need for GGPP for phytosynthetic pigments but more specifically the early production of *ent*-kaurenoic acid for use in phytohormone biosynthesis. Indeed, as discussed above this provided the origin of the plant TPS family and, hence, more specialized terpenoid metabolism throughout the spermatophytes (seed plants). Highlighted here is a recent report that provided further insight into the early evolutionary history of this key enzymatic family, particularly as this included predictive use of motifs hypothesized to be specific to the *ent*-CPP and *ent*-kaurene synthases conserved for phytohormone biosynthesis, which were identified despite the repeated derivation of these enzymes to more specialized diterpenoid metabolism in a plant lineage specific manner. Related insights into at least class II diterpene cyclase enzymatic structure–function relationships provide additional predictions, verification of which will enable more confident bioinformatic suggestions for biochemical activity of such enzymes more broadly. With regards to the subsequently acting CYPs it appears that different subfamilies have been recruited to diterpenoid biosynthesis in distinct plant lineages as well, again of potential use in investigating such metabolism. Relatively little is known about even further downstream tailoring enzymes, offering a fertile field for future investigation – *e.g.*, the installation of nitrogen in diterpenoid alkaloids remains completely opaque. Finally, the importance of more specialized diterpenoid metabolism is highlighted by the assembly, as well as subsequent lateral gene transfer, of associated BGCs, investigation of which appears to support co-inheritance as the primary driving force, although this is another area ripe for further studies.

## 7 Conflicts of interest

There are no conflicts to declare.

## Supplementary Material
